# Chronic traumatic encephalopathy in a female ex-professional Australian rules footballer

**DOI:** 10.1007/s00401-023-02610-z

**Published:** 2023-06-30

**Authors:** Catherine M. Suter, Andrew J. Affleck, Alan J. Pearce, Reimar Junckerstorff, Maggie Lee, Michael E. Buckland

**Affiliations:** 1 Department of Neuropathology, Royal Prince Alfred Hospital, Camperdown, NSW Australia; 2 School of Medical Sciences, University of Sydney, Camperdown, NSW Australia; 3 College of Science, Health and Engineering, La Trobe University, Melbourne, VIC Australia; 4 Forensic Pathology and State Mortuary Service, Nedlands, WA Australia

To the Editor:

Chronic traumatic encephalopathy (CTE) is a neuropathological diagnosis of degenerative brain disease characterized by neuronal accumulation of hyperphosphorylated tau (p-tau) around blood vessels at the depths of cortical sulci, with or without thorn-shaped astrocytes [2]. Prior exposure to traumatic brain injury (TBI; concussive and sub-concussive) is the only known risk factor for CTE [7]. CTE has been found most frequently in the brains of deceased athletes who had participated in contact sports such as football and boxing. Less commonly it has also been described in individuals with a history of repetitive head trauma sustained through other means, such as seizures, head banging behavior, violence, or during military service (reviewed in [5]). In sport, CTE risk appears dose dependent; increasing career length (and, thus, potential for exposure to TBI) correlates with a greater risk of developing CTE, and more severe CTE [6].

Contact sports in which head injuries occur commonly (and also where CTE is most frequently reported) are historically male dominated, and this likely underlies the strong male bias in CTE prevalence to date. However, the last two decades have seen a rise in popularity and participation in women’s contact sports, particularly among younger women aged 15–34 years [1]. Available evidence indicates that females are more susceptible to sports-related concussion than males, even when participation is controlled for [4]. At present, there have been only a handful of CTE cases reported in females [5], and none have derived from professional athletes. Here, we report the first case of CTE in a former professional female footballer.

The woman was in her late twenties when she died, and her death is subject to an ongoing coronial investigation. Due to the circumstances surrounding the death, it is suspected the woman died by way of suicide. There was no known history of alcohol or non-prescription drug abuse, and she had not exhibited any signs of depression or unusual behavior in the months leading up to her death. She was an avid footballer from 5 years of age, with a total contact sport career length of 18 years. She played across two codes, Australian rules football and rugby league, and participated in both simultaneously for approximately 2 years. By her mid-teens, she had progressed to playing representative women’s Australian rules football, before entering that sport professionally in her early twenties. She retired after one professional season due to musculoskeletal injury. She had suffered one diagnosed concussion, with four other possible concussions not formally diagnosed but suspected by family. She had served in the military for 9 years and participated in amateur martial arts for three years, although no concussions were reported from these activities. The family donated her brain to the Australian Sports Brain Bank (www.brainbank.org.au) where analysis took place.

The fresh brain was fixed in 15% formalin for two weeks prior to examination and standard sampling [2]. Macroscopically, the brain was unremarkable, apart from a posteriorly fenestrated septum pellucidum. Microscopically, there was a small (~ 5 mm) incidental venous angioma in the subcortical white matter of the right frontal lobe.

**Fig. 1:**
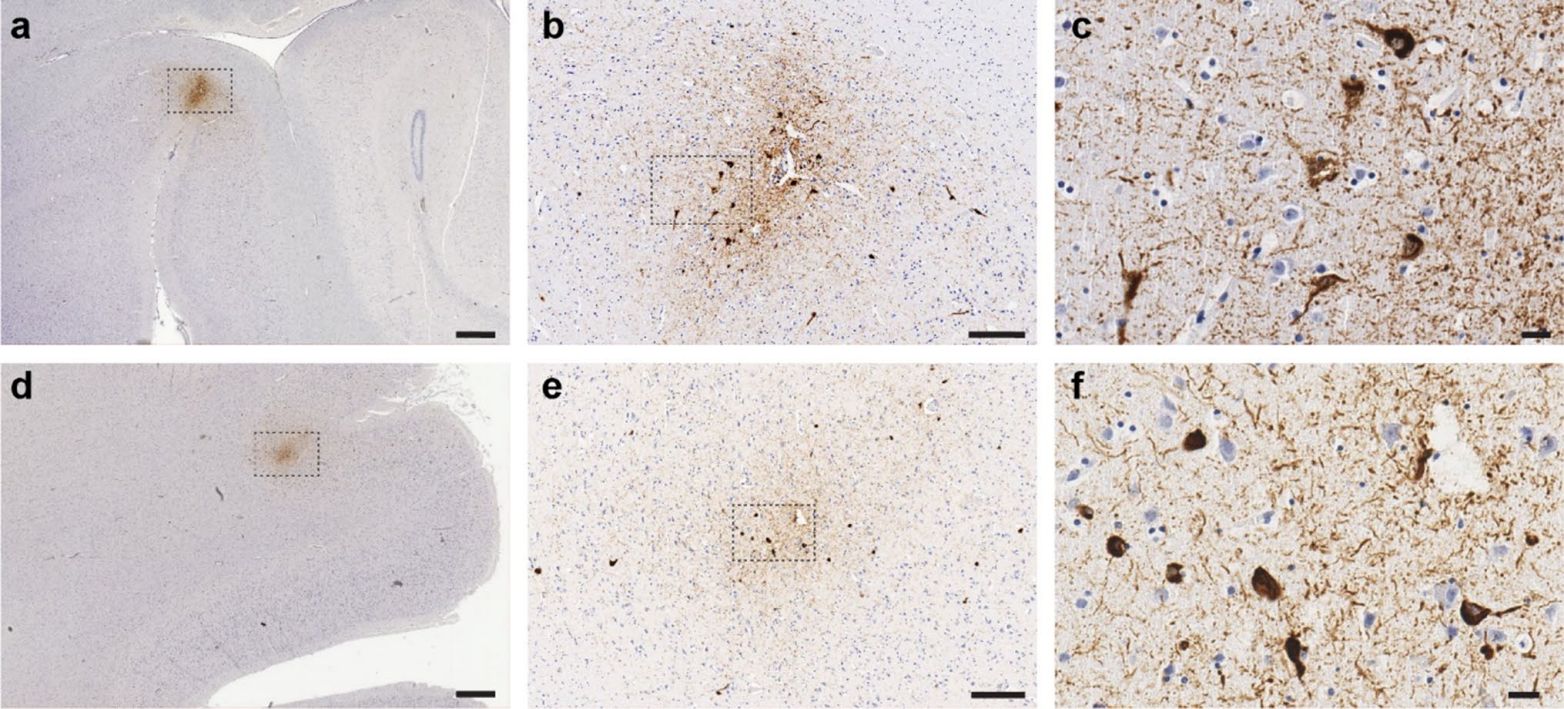
Representative CTE lesions visualized by p-tau (AT8) immunostaining. CTE lesions identified in the left rhinal sulcus (**a**–**c**) and right middle and inferior frontal gyri (**d**–**f**). Scale bars: **a**, **d** = 1 mm, **b**, **e** = 200 µm, **c**, **f** = 20 µm

Immunohistochemistry for tau (AT8 clone, Invitrogen; 1:800) revealed three foci of neuronal and neuritic p-tau in a perivascular distribution at the depths of cortical sulci (Fig. 1). The lesions were each ~ 2–3 mm in cross section. Two were found at the sulcal depths of the right frontal lobe, and another was identified in the left inferior temporal lobe (rhinal sulcus). Garvey silver staining on adjacent tissue sections of the frontal lesions demonstrated that some tau pathology was present as mature neurofibrillary tangles. In addition to the CTE foci, low-level p-tau immunoreactivity was seen in scattered neurons and/or neurites in other cortical blocks from frontal and temporal lobes. Occasional neuronal and neuritic tau staining was present in locus coeruleus and substantia nigra, while medial temporal lobe structures, basal ganglia and cerebellum were negative for neuronal tau. Immunohistochemistry for beta-A4, alpha-synuclein, and pTDP-43 showed no additional pathology. Together, these neuropathological findings fulfill current diagnostic criteria for low-stage CTE [2].

Given the positive relationship between career length and CTE risk, those who begin playing contact sport at a young age and continue playing into adulthood carry the greatest risk. There has been a significant increase in women's participation in contact sports over the past decade with the establishment of professional women's leagues in Australian Rules football, rugby, and soccer. This has led to a surge in participation, and more women playing at an elite level than ever before. This report may, thus, represent a sentinel case: as the representation of women in professional contact sports is growing, it seems likely that more CTE cases will be identified in female athletes. Given females’ greater susceptibility to concussion, there is an urgent need to recognize the risks, and to institute strategies and policies to minimize traumatic brain injuries in increasingly popular female contact sports.

While there are insufficient data to draw conclusions on any association between CTE and manner of death, suicide deaths are not uncommon in the cohorts where CTE is sought at autopsy (e.g., [8]). There is no definitive clinical syndrome ascribed to the pathology, however mood and behavioral problems are frequently described in those later found to have CTE, particularly in those who are younger at death [3]. While the case presented here did not exhibit any obvious symptoms, it nevertheless illustrates the need to consider forensic examination of the brain in suicide deaths where there is a history of repetitive head trauma.
